# Closely-related *Photobacterium* strains comprise the majority of bacteria in the gut of migrating Atlantic cod (*Gadus morhua*)

**DOI:** 10.1186/s40168-019-0681-y

**Published:** 2019-04-17

**Authors:** Typhaine Le Doujet, Concetta De Santi, Terje Klemetsen, Erik Hjerde, Nils-Peder Willassen, Peik Haugen

**Affiliations:** Department of Chemistry and the Center for Bioinformatics (SfB), Faculty of Science and Technology, UiT The Arctic University of Norway, N-9037 Tromsø, Norway

**Keywords:** Atlantic cod, Skrei, Piscivorous, Microbiota, Microbiome, Allochthonous, Metagenome-assembled genomes (MAGs)

## Abstract

**Background:**

The population of Atlantic cod (*Gadus morhua*), also known as Northeast Arctic cod, migrating Atlantic cod, or simply “skrei,” lives mainly in the Barents Sea and Svalbard waters and migrates in annual cycles to the Norwegian coast in order to spawn eggs during late winter. It is the world’s largest population of Atlantic cod, and the population is distinct from the Norwegian coastal cod (or “fjord” cod). Despite the biological, economic, and cultural importance of migrating Atlantic cod, current knowledge on the associated microbiota is very limited. Using shotgun metagenomics and metaproteomics approaches, we present here the gut microbiota, metagenome-assembled genomes (MAGs) of the most abundant bacterial species, DNA-based functional profile, and the metaproteome of Atlantic cod specimens caught at a spawning area in an open ocean outside of Tromsø, Norway.

**Results:**

Our analyses identified 268 bacterial families in DNA isolated from feces of 6 individual migrating Atlantic cod. The most abundant family was *Vibrionaceae* (52%; 83% if unclassified reads are excluded), with *Photobacterium* (genus) representing the vast majority. The recovery of metagenome-assembled genomes provided further details and suggests that several closely related *Photobacterium* strains from the *Photobacterium phosphoreum* clade are the most abundant. A genomic-based functional profiling showed that the most abundant functional subsystems are “Carbohydrates”; “Amino Acids and Derivatives”; “Protein Metabolism”; “Cofactors, Vitamins, Prosthetic, Groups, and Pigments”; and “DNA Metabolism,” which is in agreement with other studies of gut microbiomes of marine organisms. Finally, the MS-based metaproteomic dataset revealed that the functional category “Protein Metabolism” is highly overrepresented (3×) when compared to the genome-based functional profile, which shows that ribosomal proteins are rich in the bacterial cytosol.

**Conclusion:**

We present here the first study of bacterial diversity of the gut of migrating Atlantic cod using shotgun sequencing and metagenome-assembled genomes (MAGs). The most abundant bacteria belong to the *Photobacterium* genus (*Vibrionaceae* family). We also constructed functional profiles of the gut microbiome. These may be used in future studies as a platform for mining of commercially interesting cold-active enzymes.

**Electronic supplementary material:**

The online version of this article (10.1186/s40168-019-0681-y) contains supplementary material, which is available to authorized users.

## Background

Different populations of Atlantic cod (*Gadus morhua*) inhabit the oceans [1]. They show different characteristics in terms of size, surrounding habitats, migration patterns, and feeding. The Northeast Arctic cod represents the largest population of Atlantic cod. It is commonly named as the mature or migrating Atlantic cod or “skrei” [2]. The migrating Atlantic cod differs from the coastal cod by its migration pattern and its piscivorous diet (mostly consisting of herring and capelin). It spends the majority of its life in the Barents Sea and Svalbard waters but annually migrates south to spawn (for the map, see Additional file 1). A fish can measure up to 169 cm with a maximum weight of 55 kg and is usually fully grown at the age of 8 to 10 years [2, 3]. Impressively, the Atlantic cod represents approximately twice the combined biomass of Greenland halibut, haddock, deep-sea redfish, long rough dab, and thorny skate, all found in the Barents Sea [2, 3]. In late winter, at the end of January, mature Atlantic cod migrates to the Norwegian coast, as far south as Møre county and as far north as Troms and Finnmark counties. The Barents Sea and Svalbard waters make up the mature Atlantic cod nursery and feeding space for the major part of their lives [2, 3].

A study by Ghigliotti et al. [4] established the karyotypes of Atlantic cod based on 39 fish collected from 4 different locations (Norwegian coastal area and offshore). The data was in agreement with a genome consisting of a diploid number of 46 chromosomes. Moreover, a draft genome of 1 male individual (gadMor1), based on 454 sequencing, showed that the genome has an estimated size of 830 Mb [5]. A total of 22,154 genes were identified. Later, the genome quality was improved by combining several sequencing technologies and assembly tools, and the current version (gadMor2) has an estimated size of 643 Mbp and 23,243 genes [6].

The distinct life cycle, migration pattern, feeding resources, and economic and cultural importance make the migrating Atlantic cod an interesting subject for microbiota/microbiome studies, and some of these characteristics may contribute to the creation of unique and distinct bacterial communities in this type of Atlantic cod compared to other cod populations [7]. In addition, today, the intestine is considered one of the major organs in fish that interact with the environment and have important roles for the host (i.e., digestion processes, synthesis of digestive enzymes, modulation of cholesterol synthesis, etc.) [7, 8]. The intestinal microbiota of Atlantic cod was first characterized using classical microbiology methods [9–11]. These studies established that starvation and stress can have dramatic effects on the intestinal microbiota, and have provided insights into culturable microorganisms. New high-throughput methods have more recently provided data on the unculturable bacteria. Star et al. [12] determined the microbial diversity of the intestines of coastal Atlantic cod from the Oslo fjord in Norway using amplicon sequencing. They found that the gut microbiome is dominated in numbers by *Vibrionales* (50%), *Bacteriodales* (17%), *Erysipelotrichales* (4%), and *Clostridiales* (4%).

In this study, we characterized the gut microbiota of six representative migrating Atlantic cod using shotgun metagenome sequencing. By recovering the metagenome-assembled genomes (MAGs), we aimed at identifying the most abundant bacteria at a detailed taxonomic level (i.e., species or strain). Finally, we performed functional profiling of the gut microbiome to establish a platform for future mining of commercially interesting enzymes and compared this to a metaproteomics dataset.

## Results

### Sampling and total DNA sequencing

Total DNA from feces of 20 migrating Atlantic cod caught outside of Tromsø, Norway (latitude 70.018056 and longitude 18.110333) (Additional file 1) was extracted using the FastPrep®-24 system (MP Biomedicals). Six of the DNA samples passed our minimum criteria for DNA sequencing (average fragment size ≥ 440 bp, DNA concentration ≥ 1 ng/μL) and were subjected to library preparation followed by 300 bp paired-end sequencing using an Illumina Miseq instrument and the MiSeq Reagent kit v.3 (600 cycles) (see Table 1). The relatively low success rate in DNA isolation exemplifies the technical challenges that often occur when DNA is isolated from challenging samples collected from the environment (i.e., feces from fish and other animals). Sequencing statistics are provided (Additional file 2). In short, approximately 15 million reads with an average length of 266 bp were produced from each sample. After quality control and trimming of raw reads, approximately 12 million reads were kept per sample (see the “Methods” section) before further analyses.

**Table 1:** Characteristics of the six individual migrating Atlantic cod that were sequenced using shotgun sequencing with Illumina MiSeq instrument

Sample ID	Sex	Fish weight (kg)	Fish length (cm)	Digestive tract (g)	Feces weight (g)	Feces color	Feces consistency	Other obs^1^
MBRG-29	M	9.87	101	394	29.2	Dark yellowish green	Liquid mucus	Parasites
MBRG-30	F	12.07	108	381	20.0	Dark brown	Semi-solid	
MBRG-35	M	7.54	92	283	33.5	Light brownish green	Semi-solid	
MBRG-36	M	10.79	105	333	15.8	Military green	Mucus	
MBRG-38	F	10.81	99	414	23.1	Light brown	Mucus	
MBRG-44	F	11.02	97	580	81.7	Gray to light green	Liquid	

Characteristics of the fish and feces that were sequenced with 300 bp paired-end sequencing using an Illumina Miseq instrument and the MiSeq Reagent kit v.3 (600 cycles). This table shows the ID of each fish used for the study and information about migrating Atlantic cod including the sex, weight and length, and information about the extracted feces including the weight of digestive tract and feces, feces color, and consistency

^1^The presence of parasite was recorded under “other observations”

### *Vibrionaceae* makes up > 50% of the fecal microbiome

To establish the microbial community structure in fecal samples, we used the Kaiju software [13] in combination with a merged MarDB/MarRef database. The Mar databases are specific to marine bacterial genomes [14] and generate a greater proportion of reads classified and assigned to a bacterial family compared to the same analysis using RefSeq (see Additional file 3). When using Kaiju, the taxonomic classification is based on the translated sequence reads (amino acid queries) against translated databases. A rarefaction curve analysis (Additional file 4) demonstrated that enough data were available in order to identify the microbial diversity and that more data would probably lead to the discovery of few (or no) additional families since the majority of families are found after analysis of 1-2 million (of approximately 6 million) sequences.

**Fig. 1:**
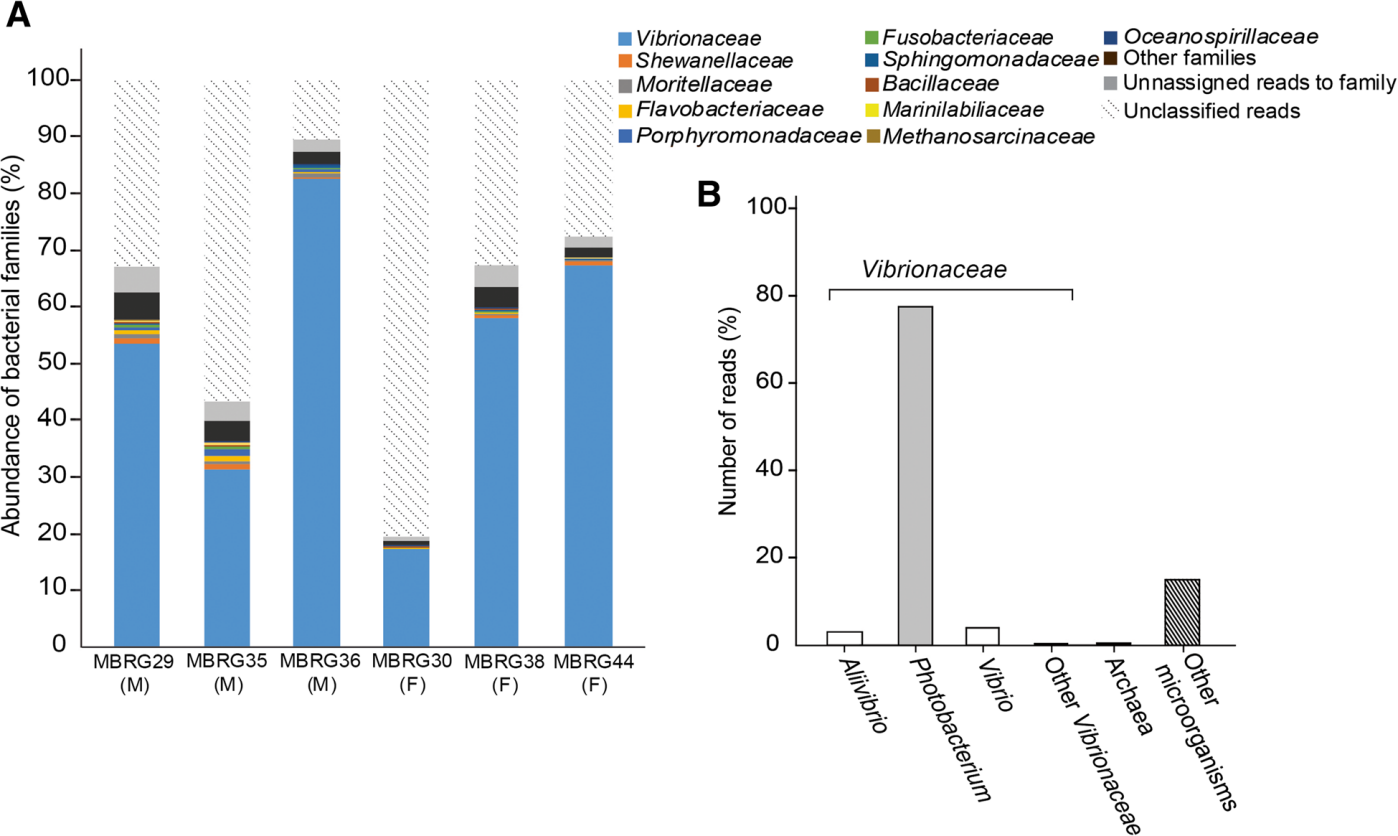
Graphical representation of the microbial diversity at the family level in the gastrointestinal tract of migrating Atlantic cod and its core microbiota across all studied samples. **a** Bar charts were constructed for visualizing the identified 11 most abundant families in 6 fecal samples including 3 males (MBRG-29, MBRG-35, and MBRG-36) and 3 females (MBRG-30, MBRG-38, and MBRG-44) after shotgun sequencing using an Illumina Miseq instrument with V3 chemistry kit (300 bp end-pair reads). **b** Bar chart of the most abundant genera across all 6 samples (core microbiota) as derived from Megan 6

A graphical representation of the microbial diversity in the gut of 6 migrating Atlantic cod at the family level is shown (Fig. 1a). With an average of 52% (83% if unclassified reads are excluded), the majority of sequence reads represent bacteria from the *Vibrionaceae* family. Other families are present at low abundance, including *Shewanellaceae*, *Flavobacteriaceae*, *Moritellaceae*, *Porphyromonadaceae*, and *Fusobacteriaceae* (0.6%, 0.4%, 0.3%, 0.3%, and 0.2% on average, respectively). On average, 268 families were detected per sample, which again demonstrates that families other than *Vibrionaceae* are found in very small numbers. Unclassified reads varied from 11 to 81% (average of 40%), which means that families not recognized by the databases could potentially be present in high numbers in fecal samples without being detected. Why the number of unclassified reads varies dramatically between samples is unclear. Nevertheless, in spite of this uncertainty, the overall picture (the diversity of families) is very similar across samples, which suggests that the unclassified reads are not causing major problems in the analysis.

Figure 1b shows the core microbiota, which simply describes the genera found across all samples. Overall, *Photobacterium* represents 78% of all genera and 91% of genera from within the *Vibrionaceae* family. In total, *Vibrionaceae* represents 84.8% of the core microbiota. “Other” microorganisms and *Archaea* represent 14.9% and 0.3%, respectively.

In conclusion, our main finding is that *Vibrionaceae*, and, in particular, representatives from the *Photobacterium* genus, dominates in numbers in the gut of migrating Atlantic cod. This is in agreement with the studies of other gut microbiota from marine carnivore fish [8]. For example, in the gut of *Lutjanus bohar* (the two-spot red snapper), bacteria from *Vibrionaceae* greatly outnumber those from other families and represent almost 70% of the total bacterial community. Overall, *Vibrionales* (which only includes the *Vibrionaceae* family) comprised a median of 69.8% of the total sequence reads from the analyzed gut communities of marine fish.

### Metagenome-assembled genomes identify the most abundant *Photobacterium* lineages

To more accurately classify the most abundant *Photobacterium* lineages in the fecal microbiome, we next assembled the metagenomics sequence data (overlapping reads) of each individual sample into longer contigs using MetaSPades v.3.10.0 [15] and then used Maxbin v.2.2.4 [16] to separate the contigs into bins based on the tetranucleotide frequency and coverage levels. For details on the analysis workflow, see the “Methods” section. Individual bins represent metagenome-assembled genomes (MAGs), which can be used to identify bacterial species and strains, and potentially help in deciphering their biological roles.

Table 2 displays the information for each of the obtained bins including the relative abundance of bins within the sample, the number of contigs, genome size, GC content, completeness and contamination of assemblies, bacterial ID from sendsketch (i.e., the two closest matches in the RefSeq database), % KID (*k*mers that match between the query and the reference), and KWID (% KID that was normalized to the genome size). In total, Maxbin produced 25 bins across all samples. Of these, 6 were discarded due to the low Maxbin completeness scores (< 50%). The remaining 19 bins were assessed for completeness and contamination using BUSCO v.3 [17] and CheckM v.1.0.11 [18]. Based on the numbers from the CheckM and the strict criteria that are typically used for evaluating the quality of MAGs [19], 4 of the MAGs can be regarded as “medium” quality (medium quality ≥ 50% completeness/< 10% contamination) and the remaining 15 as “low” quality. Obtaining higher quality MAGs would require considerable more genome refinement efforts, which was not within the scope of this study. In summary, 19 bins were kept (low and medium draft MAGs) and subjected to downstream taxonomic classification using the sendsketch script (part of BBMap package) [20].

**Table 2:** Overview of the 19 recovered MAGs (bins)

Sample ID	MAXBIN						BUSCO		CheckM		SENDSEKTCH		
	Bin ID	Rel abund^1^ (%)	Contigs (*n*)	Comp^2^ (%)	Genome size (bp)	GC content (%)	Comp^2^ (%)	Cont^3^ (%)	Comp^2^ (%)	Cont^3^ (%)	Bacteria IDs	KWID (%)	KID (%)
MBRG-29	1	63.7	803	96.3	3,744,967	39.9	91.20	14.20	96.46	34.53	*P. carnosum*/*P. iliopiscarium*	64.65/31.51	53.05/17.56
	2	14.2	1615	86.0	6,851,229	39.5	79.10	3.40	90.29	36.35	*P. phosphoreum*/*P. iliopiscarium*	32.75/9.44	14.93/9.36
	3	12.8	1495	76.6	2,903,426	33.1	53.40	1.40	74.00	20.79	*P. carnosum*/*P. iliopiscarium*	3.23/1.69	2.06/0.73
	4	6.0	1617	51.4	3,222,164	46.4	36.50	6.10	38.13	8.39	*A. logei*/*P. iliopiscarium*	1.36/1.62	0.94/ 0.78
	5	3.3	4368	92.5	10,028,463	39.0	85.80	15.50	96.08	71.43	*A. salmonicida*/*A. logei*	66.92/49.50	28.22/23.22
MBRG-30	1	91.6	682	97.2	5,125,278	39.9	97.30	5.40	97.78	12.42	*P. iliopiscarium*/*P. carnosum*	74.91/21.50	56.58/19.27
	2	8.4	30,288	50.5	48,009,385	46.2	39.20	2.70	63.83	18.65	*A. salmonicida* 2FI238/*A. logei*	34.35/26.11	3.07/2.60
MBRG-35	1	12.3	181	89.7	2,762,496	40.7	93.90	2.00	70.29	0.92	*P. iliopiscarium*/*P. carnosum*	77.02/21.72	31.56/13.18
	2	6.5	400	96.3	4,937,125	38.8	87.90	3.40	95.67	17.85	*P. iliopiscarium*/*P. carnosum*	33.45/8.50	24.40/7.89
	3	2.1	496	78.5	1,221,145	31.2	60.80	0.0	78.28	1.12	*P. iliopiscarium*/*P .carnosum*	3.49/0.86	0.65/0.24
	4	1.1	974	62.6	1,948,287	34.4	39.20	11.50	60.05	28.35	*P. carnosum*/*P. iliopiscarium*	0.80/0.72	0.34/0.21
	5	0.5	5824	83.2	9,939,730	41.7	63.50	11.50	79.55	41.82	*A. wodanis*/*A. sp* 1S128	6.71/6.56	5.75/2.92
MBRG-36	1	58.0	59	78.5	1,315,797	41.9	80.40	0.0	48.40	0.16	*P. phosphoreum*/*P. sp*	96.24/9.85	8.76/3.01
	2	1.6	544	61.7	3,480,193	32.3	43.90	0.70	44.64	0.10	*P. carnosum*/*P. iliopiscarium*	3.96/2.31	0.95/0.38
	3	1.4	1394	94.0	4,378,218	38.1	81.10	11.50	93.10	42.38	*P. iliopiscarium*/*P. carnosum*	1.36/1.01	0.88/0.96
	4	1.2	1348	86.9	3,322,850	45.4	70.90	8.10	81.40	9.39	*P. iliopiscarium*/*P. phosphoreum*	1.19/1.00	0.50/0.27
	5	1.1	938	73.8	2,776,503	42.7	64.20	9.50	84.89	19.86	*A. salmonicida*/*A. logei*	0.32/0.25	0.21/0.15
MBRG-38	1	80.1	513	96.3	3,685,334	40.3	86.40	20.90	96.49	43.52	*P. carnosum*/*P. iliopiscarium*	64.15/17.82	34.85/14.27
MBRG-44	1	75.4	109	85.0	2,061,831	40.6	87.80	0.0	63.32	0.16	*P. iliopiscarium*/*P. carnosum*	85.02/22.88	25.91/10.28

The information for each of the obtained MAGs include the relative abundance of the bins; number of contigs; genome size; GC content; completeness and contamination of assemblies using Maxbin, BUSCO, and CheckM; bacterial ID from sendsketch (i.e., the two closest matches in the RefSeq database); % KID (*k*mers that match between the query and the reference); and KWID (% KID that was normalized to the genome size)

^1^The abundance of bins within a sample by Maxbin

^2^The completeness of genome by Maxbin, BUSCO, and CheckM.

^3^The contamination score using BUSCO and CheckM for each MAGs

**Fig. 2:**
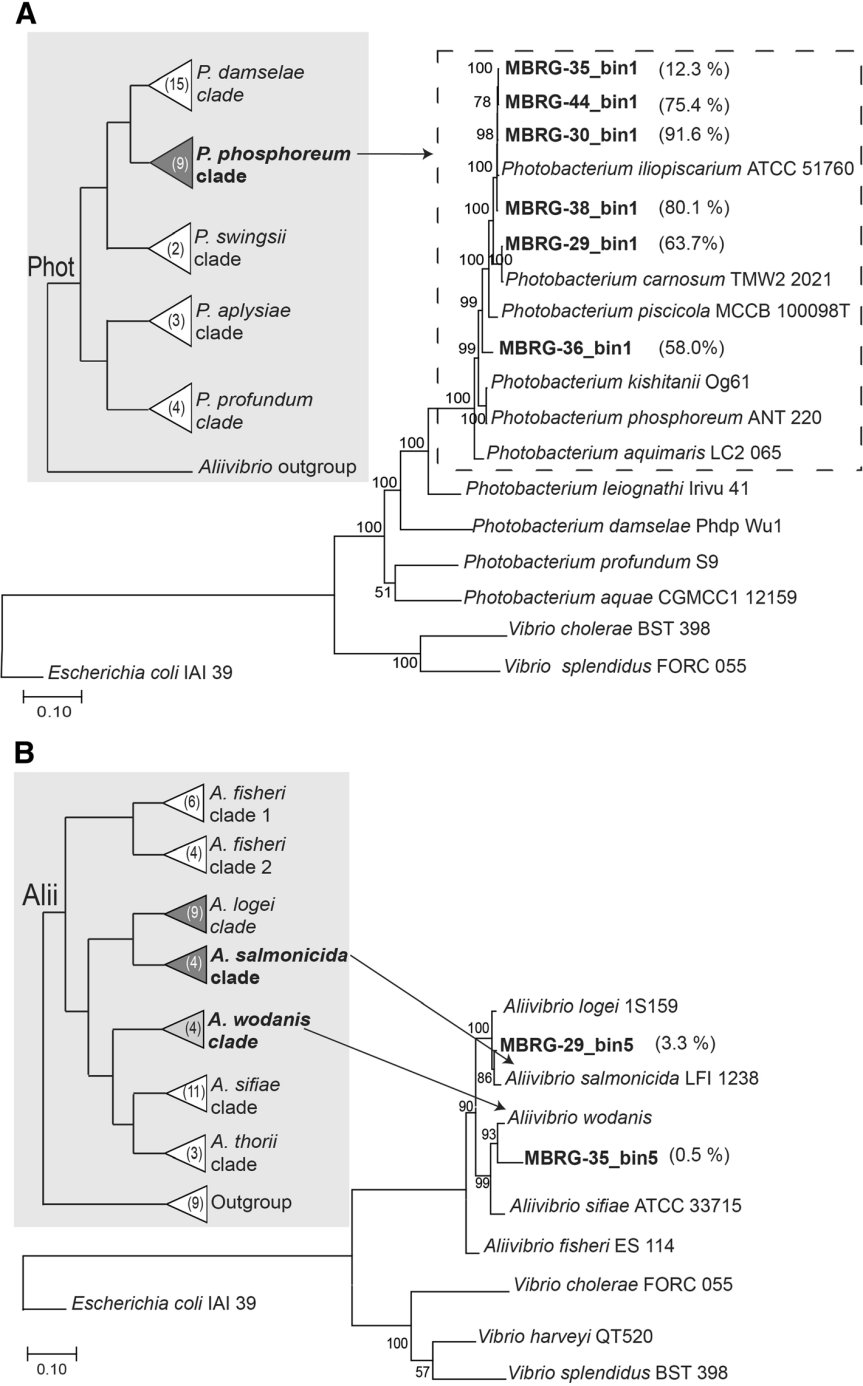
Maximum likelihood trees based on datasets of marker genes as identified by ezTree. **a** The phylogram is based on a dataset of the most abundant bin from each fecal sample and *Photobacterium* reference genomes [39]. The tree was rooted in the 2 *Vibrio* and *Escherichia* genomes. Bootstrap values are from a ML analysis (500 pseudoreplicates, JTT model). The cladogram shown in gray background is from Hilgarth et al. [39] and shows the established phylogenetic relationships between a wider range of *Photobacterium* species. The numbers in parentheses represent the number of species in each clade. **b** The phylogram is based on a dataset that includes MBRG-29_bin5 and MBRG-35_bin5 and reference genomes from the *Aliivibrio* genus. The cladogram is from Ast et al. [40]

The results from sendsketch support that the most abundant bin in each sample represents, as expected, bacteria from the *Photobacterium* genus, more specifically bacteria from the *P. phosphoreum* clade based on the best hit from the RefSeq database (see cladogram in Fig. 2a). Four bins (i.e., MBRG-29_bin5, MBRG-30_bin2, MBRG-35_bin5, and MBRG-36_bin5) with low abundance represent bacteria from the *Aliivibrio* genus, three of which are from the *Aliivibrio salmoinicida*/*Aliivibrio logei* sister clades and one from the *Aliivibrio wodanis* clade (see cladogram in Fig. 2b). The identity of MBRG-29_bin4 is unclear.

**Fig. 3:**
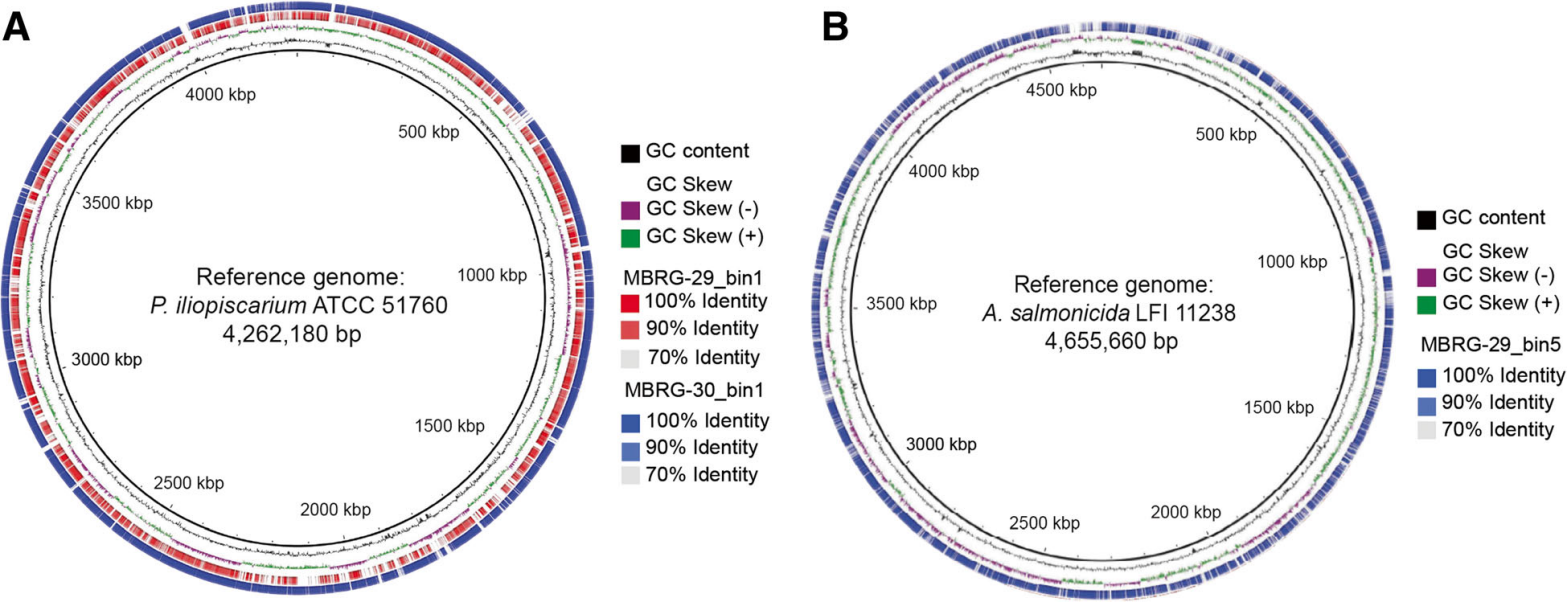
Comparison of the recovered MAGS (bins) and downloaded reference genomes. **a** Comparison between the two recovered MAGs (bins) and the *P. iliopiscarium* ATCC-51760 reference genome. **b** Comparison between one recovered MAG (bin) and the *A. salmonicida* LFI1238 reference genome. The figure was generated using the BLAST Ring Image Generator (BRIG) tool. The reference is shown as a black ring at the center, GC content and GC skew are displayed, and matches between MAGs and the reference are shown as colored rings scaling from high to lower intensities corresponding to 100%, 90%, and 70% identity

To ascertain the identity of the most abundant bin from each sample, we next used EzTree to identify the single-copy markers genes suitable for inferring phylogenies and then used MEGA7 [21] to construct a maximum likelihood (ML) tree based on the concatenated dataset identified by EzTree [22]. Reference strains from the *Photobacterium* genus were added to the dataset. The topology of the resulting ML phylogeny (Fig. 2a) is strongly supported by high bootstrap values and provides further support for the placement of the six most abundant MAGs within the *P. phosphoreum* clade, but with slightly different affinity to different species. MBRG-35_bin1, MBRG-44_bin1, MBRG-30_bin1 and MBRG-38_bin1 group with *Photobacterium iliopiscarium*, MBRG-29_bin1 with *Photobacterium carnosum*, and finally, MBRG-36_bin1 is placed between *P. phosphoreum* and *Photobacterium piscicola*. The latter might suggest the presence of a hitherto undescribed species. MBRG-29_bin1 and MBRG-30_bin1 mapped to a reference genome (*P. iliopiscarium*; assembly number: GCA_000950265.1) are shown in Fig. 3a. Both bins cover most of the reference with high identity (≥ 90%).

In a similar approach, we constructed a ML phylogeny of MBRG-29_bin5 and MBRG-35_bin5 and representative reference genomes from the *Aliivibrio* genus (Fig. 2b). The resulting tree strongly supports that MAGs MBRG-29_bin5 and MBRG-35_bin5 represent genomes from *A. salmonicida* and *A. wodanis* (or very closely related undescribed species), respectively. MBRG-29_bin5 mapped onto the *A. salmonicida* reference genome (assembly reference: GCA_000196495.1) is shown in Fig. 3b. The bin covers most of the reference with high identity (≥ 90%). In conclusion, by using metagenomics assembly and binning in combination with phylogenetics, we provide a strong support for that closely related strains from the *P. phosphoreum* clade represent the most abundant bacteria in the cod fecal microbiome. We also demonstrated the presence of low abundance strains that are closely related to *A. salmonicida* and *A. wodanis*.

### Functional profiling of the gut microbiota/microbiome of migrating Atlantic cod

**Fig. 4:**
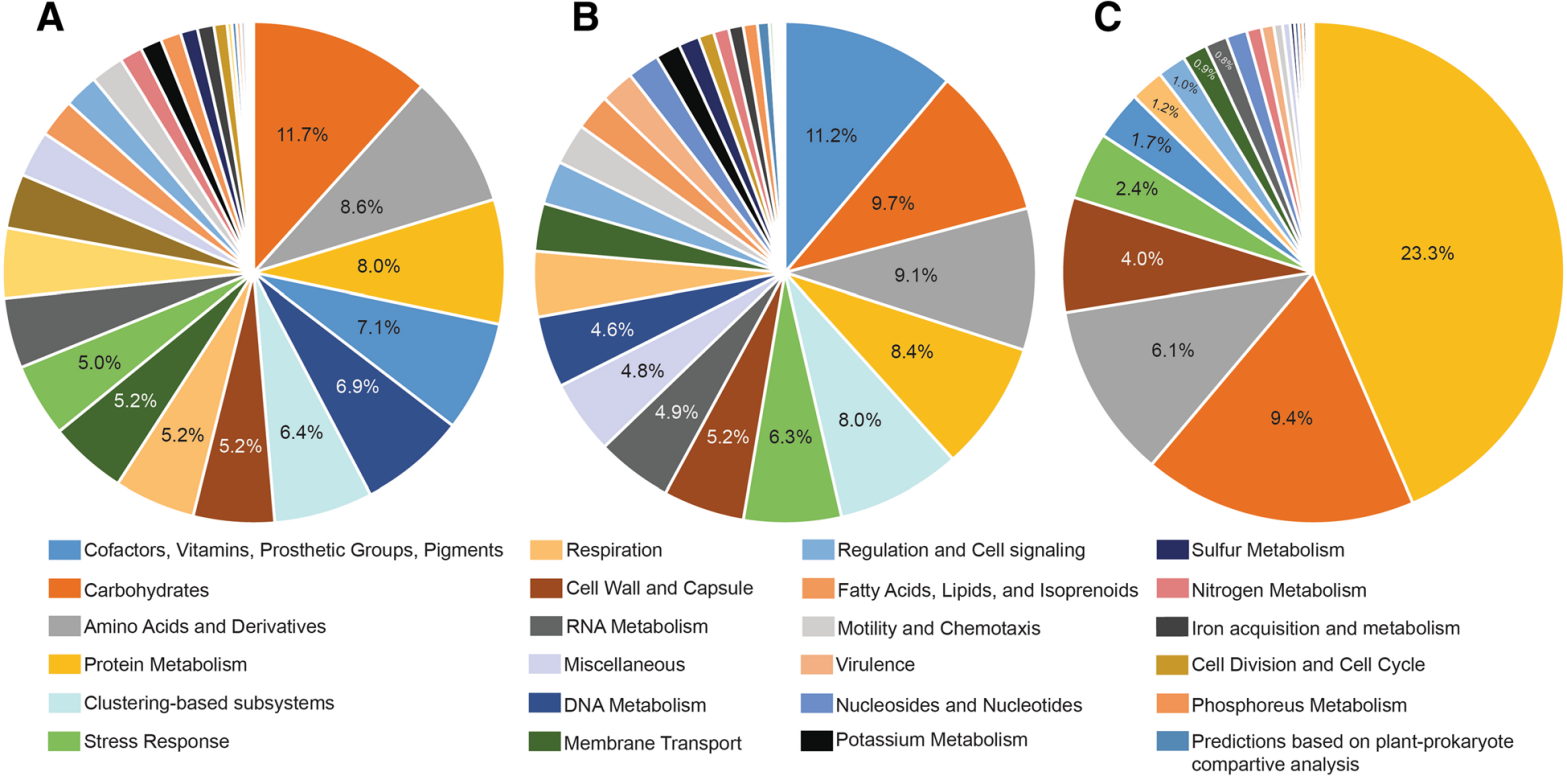
Graphical representation of the functional profiling showing the relative abundance of SEED subsystems at level 1 across six fecal migrating Atlantic cod samples, as derived from SUPER-FOCUS. **a** The pie chart is based on all unannotated sequencing reads across six fecal samples. **b** The pie chart is based on the most abundant bacteria (i.e., six most abundant bins). **c** The pie chart is based on the metaproteomics data for the six individuals

To describe the functional profile of the gut microbiome of migrating Atlantic cod, we next used SUPER-FOCUS v.0.26 [23] to assign all unannotated shotgun sequencing reads (i.e., approx. 70 million reads in total after trimming) to the SEED functional classification system at subsystem level 1 (for SEED functions/subsystem level 3, see Additional file 5). SUPER_FOCUS provides the relative abundance of each functional category. Figure 4a shows the relative abundance averaged across the 6 samples as a pie chart (see Additional file 6 for individual profiles). Here, the top 5 functional classes belong to core metabolic functions including “Carbohydrates” (11.7%); “Amino Acids and Derivatives” (8.6%); “Protein Metabolism” (8%); “Cofactors, Vitamins, Prosthetic Groups, and Pigments” (7.1%); and “DNA Metabolism” (6.9%). Same calculations were done for the most dominant bacteria (in numbers) to investigate if these have a functional profile that is unique or different from the profile originating from the total dataset (Fig. 4b; see Additional file 7 for the 6 individual profiles). This was done by selecting only 1 bin from each sample with the highest relative abundance. All bins represent bacteria from the *Photobacterium* genus (see Table 2). In general, the overall picture described above for all reads (Fig. 4a) was recovered, with a few conspicuous differences (Fig. 4b). For example, “DNA metabolism” is the tenth and fifth most abundant class for the most numerous bacteria and the entire dataset, respectively. Similarly, the top 1 category for most numerous bacteria is “Cofactor, Vitamins, Prosthetic Groups, and Pigments” instead of “Carbohydrates,” with a relative abundance of 11.2%. Interestingly, this result suggests that *Photobacterium* genomes contain a relatively rich pool of genes for producing “Cofactors, Vitamins, Prosthetic Groups, and Pigments,” compared to the entire gut microbiome of Atlantic cod. In contrast, *Photobacterium* genomes apparently contain less genes associated with “Carbohydrates,” when compared to the entire gut microbiome.

The functional profiling described above is based on the genomic data and does not provide information on what type of bacterial proteins are produced and thus present in the cod intestinal tract. To investigate the bacterial proteome, total protein preparations extracted from each of the 6 fecal samples were subjected to a MSMS analysis (performed at the Tromsø University Proteomics platform, TUPP). The analysis produced approximately180,000 MSMS spectra in most of the runs, with a subsequent identification rate ranging between 0.2 and 3% meaning that the analysis was unable to identify the vast majority of proteins in the samples. The retained proteins that matched our database (i.e., total annotated proteins in metagenomics datasets) were assigned to the SEED subsystem at level 1. Figure 4c shows how the identified proteins are distributed into SEED subsystems, when taken into account the average relative abundance of each protein (i.e., the riBAQ numbers) across the 6 samples. Despite the small fraction of identified proteins, 3 of the top 5 SEED subsystems identified in the genomic data are recovered in the proteome (i.e., “Protein Metabolism,” “Carbohydrates,” and “Amino acids and Derivatives”). However, the relative abundance of the “Protein Metabolism” category is approx. 3× higher in the proteome compared to the genome-based dataset (23.3% and approx. 8%). The “overrepresentation” of proteins from that category is due to a large number of MSMS spectra matching the ribosomal proteins in our database, which can be expected since ribosomal proteins in, for example, *Escherichia coli* account for up to 50% of the total cell dry mass [24].

## Discussion

High-throughput methods, such as next-generation sequencing (NGS), represent effective tools today for studying the gut microbiota/microbiome of, for example, fish. Most of the existing literature on this topic used amplicon sequencing, i.e., a study by Star et al. [12] characterized the microbial diversity of coastal Atlantic cod using amplicon sequencing. In the current study, we implemented shotgun sequencing to explore the gut microbiota and microbiome of six individuals of a migrating population of Atlantic cod (i.e., skrei). The total DNA was sequenced to avoid overestimation of bacterial diversity that may be observed when using 16S rDNA-based amplicon sequencing [25]. For taxonomic classification, the sequences were compared to new databases that are specific to marine bacteria (i.e., MarDB and MarRef). Importantly, our study deals with fecal matter extracted from the intestinal tract. This means that rather than describing the total bacterial community associated with the gut, the majority of the presented microbiome will represent allochthonous bacteria, i.e., free-living bacteria that are considered as transient and associated with the digesta and generally not with the mucosal surface of the gut [26].

*Vibrionaceae* was identified as the most abundant family. This finding is in agreement with other studies of the microbiota of carnivorous fish, including *Lutjanus bohar* (turbot fish) [8, 27] and coastal Atlantic cod [12]. More specifically, we identified highly abundant and closely related species or strains of *Photobacterium* from the *P. phosphoreum* clade, and we present the assembled genomes of these bacteria as derived from the shotgun data. Our data therefore offer knowledge on the Atlantic cod microbiome at a far more detailed level than previous studies. Compared to the results from coastal Atlantic cod, representatives from the orders *Bacteriodales* and *Erysipelotrichales* were not detected in migrating Atlantic cod (vs 17% and 4% in coastal cod, respectively) and representatives from *Clostridiales* are only < 1% (vs 4.9% in coastal cod). The reason for these variations between migrating and coastal Atlantic cod is currently unknown but may be due to the differences in, for example, diet, life cycle, migration pattern, habitat, etc. or simply be a result of chance or seasonal changes. Differences in sequencing methods (shotgun metagenome sequencing vs amplicon sequencing), bioinformatics tools, and choice of databases may also have contributed to the differences in the results between the two studies.

Shotgun sequencing data also offer additional advantages (i.e., compared to amplicon data), e.g., it allows for functional profiling since all genes are sequenced. An overview of the main functional SEED subsystems found in the gut microbiome is presented. In agreement with other studies of gut microbiomes of marine organisms [27, 28], “Carbohydrates”; “Amino Acids and Derivatives”; “Protein Metabolism”; “Cofactors, Vitamins, Prosthetic, Groups, and Pigments”; and “DNA Metabolism” are among the most abundant subsystems. Similar patterns have also been observed in metagenomes of soils [29]. Furthermore, we performed an MS-based proteomics experiment on the fecal samples to compare and validate the genome-based functional profile. Although the overall profile resembles the genome-based functional profile, the category “Protein Metabolism” is highly overrepresented (3×) in the proteomes, which is in agreement with ribosomal proteins accounting for up to 50% of the total cell dry mass of *E. coli* [24]. Finally, it should be stressed that the metaproteomics data that we present here must be interpreted with caution. Marine bacteria are poorly represented in current databases; thus, metaproteomics studies of marine samples will typically have limitations. For example, our data show that only 0.2-3% of MSMS spectra can be identified with high statistical support. In other words, because we are currently unable to identify the vast majority of proteins present in the cod feces, strong conclusions should not be made.

The microbiota of animal guts evolves to exploit structural features and energy resources found in the gut. Over time, genes that benefit the microbes while minimizing any negative impacts on the host are enriched. Given this assumption, the gut microbiota of migrating Atlantic cod is expected to contain bacteria with genes highly specialized for breaking down biomass consisting of materials that contain a broad range of fibrous proteins (i.e., collagen, keratin, muscles), carbohydrates (e.g., chitin), and other macromolecules originating from different marine animals. As described in the “Results” section, “Carbohydrates” subsystem at level 1 was identified at high abundance. For example, “Chitinase” was detected among the top 30 of the most occurring functions, which is in agreement with the literature showing that *Photobacterium* (i.e., the most abundant bacteria in our samples) is associated with degradation of chitin in the gut of cold water fish [26]. Chitinases and other enzymes from the cod microbiome may be exploited for biotechnological purposes, especially when cold-active enzymes are sought. In future investigations, we therefore aim at finding genes that code for hydrolytic enzymes with potential in biotechnological applications, such as in the conversion of marine biomass (e.g., underutilized side streams).

## Methods

### Sample collection

Migrating Atlantic cod were collected from a single spawning area located in an open ocean outside of Tromsø, Norway, using a fishing net (latitude 70.018056 and longitude 18.110333), at a depth of 63-108 m with a water temperature of 5 °C on 22 February 2017 (Fig. 1). This process was professionally performed by local fishermen, and the gastrointestinal tract of 20 mature Atlantic cod was transported to our laboratory facilities in Tromsø, at UiT The Arctic University of Norway. The 20 individuals included both females and males with an average weight of 9.5 kg and length of 93.8 cm (Additional file 8). Fecal matters were extracted from the entire intestine and rectal section, excluding the stomach. In order to limit the risk of cross-contamination, new gloves were used between each individual and the equipment used for extracting feces was sterilized using ethanol and a Bunsen burner. For each fish, the weight of the digestive tracks and extracted feces, color, consistency, sex, and the presence of parasites were recorded. The fecal samples were conserved at − 80 °C in 20% glycerol until isolation of metagenomics DNA, shotgun sequencing, and further bioinformatics analysis.

### DNA extraction and purification

Total DNA was extracted from fecal matter using the FastDNA^TM^ Spin Kit for soil (MP Biomedicals, USA) as follows: 200-250 mg of feces were added per E-lysing matrix tube. Six tubes were used per fish. Thus, 120 E-lysing matrix tubes were necessary for extracting the metagenomics DNA of 20 individuals. The samples were homogenized in the FastPrep®-24 system (MP Biomedicals) for 10-15 s at speed 4. For efficient lysis of the Gram-positive bacteria, 200 μL of 100 mg/mL lysozyme solution was added to 3 E-lysing matrix tubes per fish and incubated for 10 min at 37 °C. The steps using vortex were replaced by gentle hand rotation of the samples, and the DNA pellet was gently resuspended using a micropipette with the biggest tips available to minimize DNA fragmentation. The final centrifugation step (washing step with SEWS-M) was set to 2 min instead of 1 min. After total DNA extraction, the genetic material was purified overnight in 3 M, 5.2 pH sodium acetate. After 12 min of centrifugation at 14,000×*g* and 4 °C, the supernatant containing the DNA was transferred to new a tube containing 2.5 volumes of 96% ethanol. After 12 min of centrifugation at 14,000×*g* and 4 °C, the supernatant was discarded and the DNA pellet was washed using 200 μL of 70% ethanol. This washing step was repeated once. Afterwards, the DNA pellet was eluted in 80 μL clean and sterilized water. The purity of DNA and concentration was assessed using Nanodrop 2000c (ThermoFisher Scientific, USA). Finally, the DNA concentration of the pure samples was checked with precision using Qubit 2.0 fluorometer (ThermoFisher Scientific) and stored at − 20 °C until use.

### Shotgun sequencing and quality control

DNA libraries were prepared from the 20 samples by following, in general, the Nextera XT DNA Library Prep Reference Guide (Illumina, USA). However, after tagmentation, the first cleanup step of the tagmented DNA was omitted to avoid loss of DNA. After amplification of the tagmented DNA, the cleanup procedure of PCR products was slightly modified by adding only 17 μL of resuspension buffer (RSB) to each well of NAP2 plate. In order to create optimum cluster densities during a sequencing run, the DNA was precisely quantified using Qubit 2.0 Fluorometer (ThermoFisher Scientific). Next, DNA samples were quality controlled (average length of DNA fragments) using a 2100 Agilent Bioanalyzer (Agilent, USA). Six libraries passed our final criteria (DNA fragments ≥ 440 bp and 1 ng/μL). The 6 libraries were normalized to 4 nM. The final concentration of the samples and PhiX control was 15 pM. Finally, in a new tube, 594 μL of DNA library and 6 μL of PhiX control were combined and loaded onto the Miseq reagent cartridge. Subsequently, 300 bp paired-end sequencing was performed using an Illumina Miseq instrument and the MiSeq Reagent kit v.3 (600 cycles). After sequencing, sequence reads representing host DNA were removed and the quality of the sequence reads was checked using FastQC [30]. The genomic information of Atlantic cod (Gadus morhua) is available under the accession number SAMEA4028801 and SAMEA3138401. Low-quality reads were trimmed using Trimmomatic 0.36 [31] with the following parameters: SLIDING WINDOW=4:20, MINLEN=50, LEADING=3, TRAILING=3, HEADCROP=12, and AVGQUAL=20. Finally, FastQ Screen v.0.11.4 was used for confirming the absence of PhiX reads in the datasets.

### Taxonomic profiling and core microbiota

Using trimmed data, the taxonomic profiling of the six sequenced samples was performed using Kaiju [13] with default parameters. Kaiju was configured to use protein sequences from the Mar databases MarDb and MarRef version 2 [14] that are specific to marine organisms. Using kaiju outputs, a taxonomic report was produced for each sample and contained both name and percentage of assigned reads to bacterial families and the number of unclassified reads. Finally, a bar chart graph that describes the bacterial diversity of each fecal sample was manually constructed using the taxonomic reports. Next, the core microbiota was analyzed using Megan 6 [32] for determining what taxa appeared to be the most abundant across all samples. No threshold for the sample and class was set.

### Rarefaction curve

Rarefaction curves were constructed using the Kaiju output files that contained the identified bacterial families of the 6 fecal samples. First, each output file was converted into “rarefaction reports” that contain numerous lines called reads (i.e., up to 6 million) where 1 line/read represents 1 bacterial family. Thus, several lines/reads can correspond to 1 particular bacterial family. Finally, rarefaction curves were constructed using the “rarefaction report” from each individual sample where bacterial families were randomly picked up at different numbers of reads ranging from 1000 to 6 million reads. However, when a particular family was identified multiple times in a sample, this one was represented only once and as a unique family in the rarefaction curves.

### Assembly of genomes from metagenomic-assembled genomes

Genomes were assembled following an established workflow (see Additional file 9). First, the host DNA and 16S rRNA genes were removed using FastQ Screen v.0.11.4 [33] and “filter_16S_extended.py” with the command line -f -s -b -n 16 -o, respectively. Then, the files were synchronized using “repair.sh” from BBMap package [20] followed by the trimming of the low-quality reads using trimmomatic v.0.26 with the same parameters as previously done on the raw reads at the exception of HEADCROP that was set to 15. Subsequently, the overlapping reads were merged using Seqprep [34], and each metagenome was assembled individually into longer contigs with MetaSPAdes v3.10.0 [15] and binned using Maxbin v.2.2.4 [16], using default parameters. Next, the genome completeness and contamination were checked using BUSCO v.3 [17] and CheckM v.1.0.11 [18] with default parameters. Afterwards, “sendsketch” from BBMap package was used for assessing the identity of obtained individual bins. Single-copy marker genes were identified in the metagenome-assembled genomes (MAGs) and concatenated in as multiple alignments with EzTree pipeline [22] and used for constructing the phylogenetic trees with MEGA 7 [21]. Finally, BRIG v.0.95 (BLAST Ring Image Generator) [35] was used to blast MAGs onto reference genomes using nucleotide fasta files.

### Functional profiling

SUPER-FOCUS v.0.26 with default parameters [23] was used to establish the functional profiling of the fecal samples from migrating Atlantic cod using the pre-processed samples for quality check and removal of host DNA and 16S rRNA genes. On average, 12 million reads per sample were used for the identification of the 3 subsystems levels that are present in the samples by using the reduced SEED. The tool was also used on the MAGs of interest (i.e., most abundant species). SUPER-FOCUS v.0.26 also generated a file with the SEED functions present in the samples. A SUPER-FOCUS plots script was used for constructing a graphical representation of the top 30 most occurring functions in the samples using SUPER-FOCUS output file containing all subsystem level and functions of the 6 samples.

### Metaproteomics analysis

Proteins were prepared for subsequent LC-MS/MS analysis with a Thermo Scientific Q-Exactive HF-X mass spectrometer. Firstly, the six fecal samples were lysed in a 2× lysing solution (200 mM Tris-HCl pH 6.8, 50% glycerol, 4% SDS, 5% beta-mercaptoethanol, and 0.2% bromophenol blue) and heated at 95 °C for 15 min. The samples were then sonicated for 5 min and centrifuged at 14,000 rpm for 2 min before loading of the proteins into SDS gel. Next, each lane was cut into three gel pieces and subjected to in a gel reduction, alkylation, and tryptic digestion with 6 ng/μL trypsin (V511A, Promega, WI, USA) [36]. OMIX C18 tips (Varian, Inc., Palo Alto, CA, USA) were used for sample cleanup and concentration quantification. Peptide mixtures containing 0.1% formic acid were loaded onto a Thermo Fisher Scientific EASY-nLC1200 system and EASY-Spray column (C18, 2 μm, 100 Å, 50 μm, 50 cm). Peptides were fractionated using 5-80% acetonitrile gradient in 0.1% formic acid over 140 min at a flow rate of 300 nL/min. The mass spectrometry run was performed on the separated peptides for the six samples separately. A top 10 method was used to collect the data using a data-dependent mode and processed with Proteome Discoverer 2.2 software. An in-house Mascot server (Matrix Sciences, UK) was used for the search of the fragmented spectra against the in-house annotated sequences of the six samples. The peptide and fragment mass tolerance were set to 10 ppm and 0.1 Da, respectively. Finally, the peptides were identified by filtering the peptide ions using a false discovery rate (FDR) of 5% [37].

## Additional files

Additional file 1: Life cycle, migration pattern, and sampling area of the Atlantic cod population. Migrating Atlantic cod were sampled outside Tromsø, Norway (latitude 70.018056 and longitude 18.110333) at 63–108 m depth, with a sea temperature of 5 °C on February 22, 2017. A red pin indicates the collection area of Atlantic cod. Black arrows show the spawning migration routes of migrating Atlantic cod from the Barents Sea and Svalbard waters to the Norwegian coasts, during late winter. (JPG 818 kb)

Additional file 2: Sequencing statistics of the fecal samples. (XLSX 12 kb)

Additional file 3: Taxonomic comparison of fecal samples using different databases (RefSeq and the Mar databases) with Kaiju. Graphical representation of the number of detected bacterial families with A) RefSeq, B) MarRef, C) MarDB, and D) combined MarDB with MarRef. (JPG 1932 kb)

Additional file 4: Rarefaction curve analysis of the individual fecal samples. The analysis shows the number of detected families per sample as a function of the number of reads. The six samples include three males (MBRG-29, MBRG-35, MBRG-36) and three females (MBRG-30, MBRG-38, MBRG-44). (JPG 1346 kb)

Additional file 5: Functional profiling showing the top 30 most occurring SEED functions (including their corresponding subsystem level 3) of all unannotated shotgun sequencing reads from six fecal samples of migrating Atlantic cod. This figure was constructed using SUPER-FOCUS plot. (JPG 1496 kb)

Additional file 6: Functional profiling showing the relative abundance of SEED subsystems at level 1 of all unannotated sequencing reads from six fecal samples of migrating Atlantic cod, as derived from SUPER-FOCUS. (JPG 1217 kb)

Additional file 7: Functional profiling showing the relative abundance of SEED subsystems at level 1 of the most abundant bacteria in the gut of Atlantic cod (i.e., individual profiles of one bin from each sample with the highest relative abundance), as derived from SUPER-FOCUS. (JPG 4081 kb)

Additional file 8: Properties of 20 mature Atlantic cod sampled for this study in an open ocean outside of Tromsø, Norway. (XLSX 15 kb)

Additional file 9: Workflow of the nine steps for recovering metagenome-assembled genomes (MAGs) from six fecal samples of Atlantic cod. (JPG 759 kb)

## Abbreviations

GI: Gastrointestinal
MAGs: Metagenome-assembled genomes
