# Supplementary figures and images for: Closely-related Photobacterium strains comprise the majority of bacteria in the gut of migrating Atlantic cod (Gadus morhua)
Source: Microbiome. 2019 Apr 17;7:64. doi: 10.1186/s40168-019-0681-y (PMC6471968; doi:10.1186/s40168-019-0681-y)

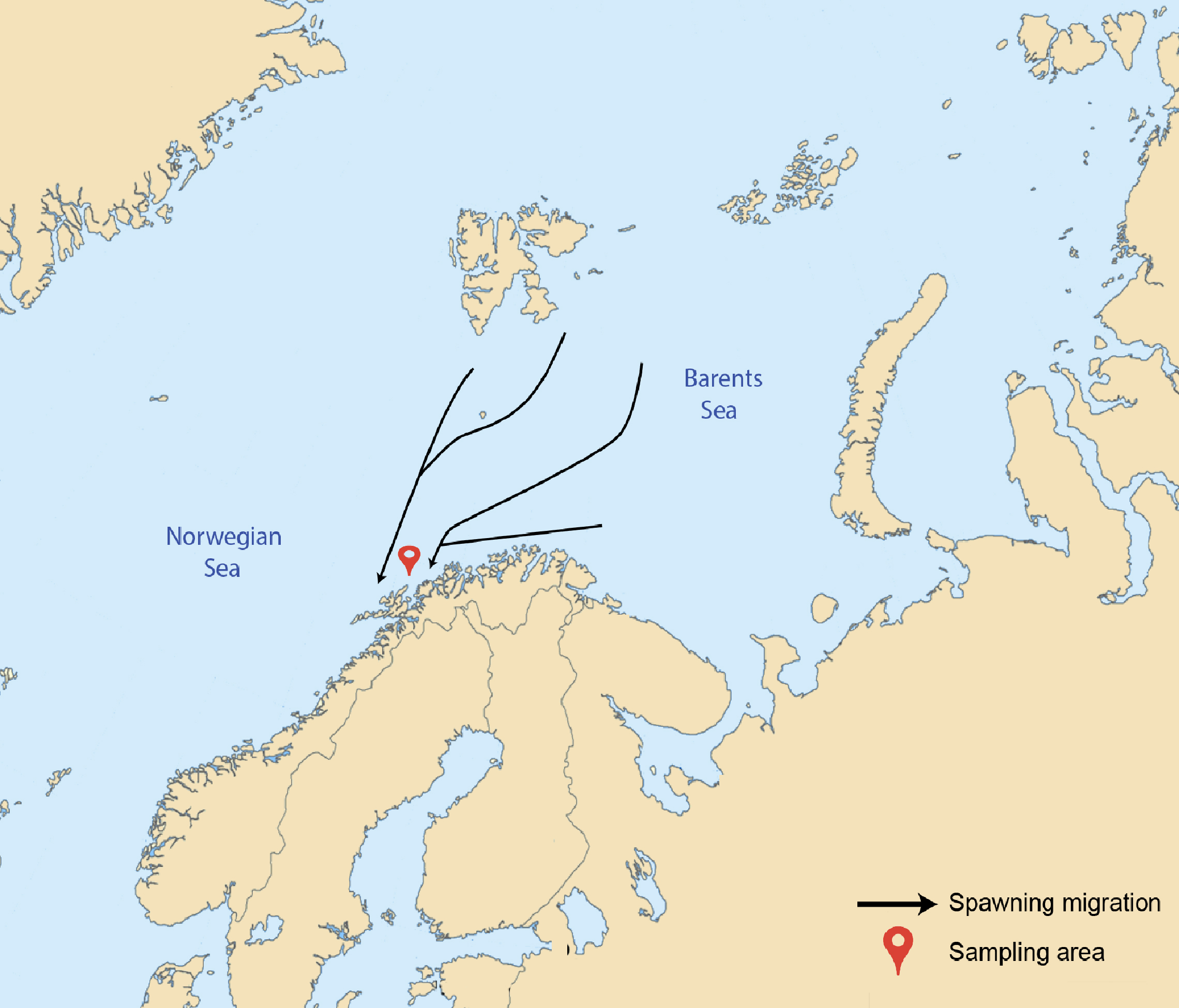

Supplement: Supplementary file 1 — Life cycle, migration pattern, and sampling area of the Atlantic cod population. Migrating Atlantic cod were sampled outside Tromsø, Norway (latitude 70.018056 and longitude 18.110333) at 63–108 m depth, with a sea temperature of 5 °C on February 22, 2017. A red pin indicates the collection area of Atlantic cod. Black arrows show the spawning migration routes of migrating Atlantic cod from the Barents Sea and Svalbard waters to the Norwegian coasts, during late winter. (JPG 818 kb) [file 40168_2019_681_MOESM1_ESM.jpg]

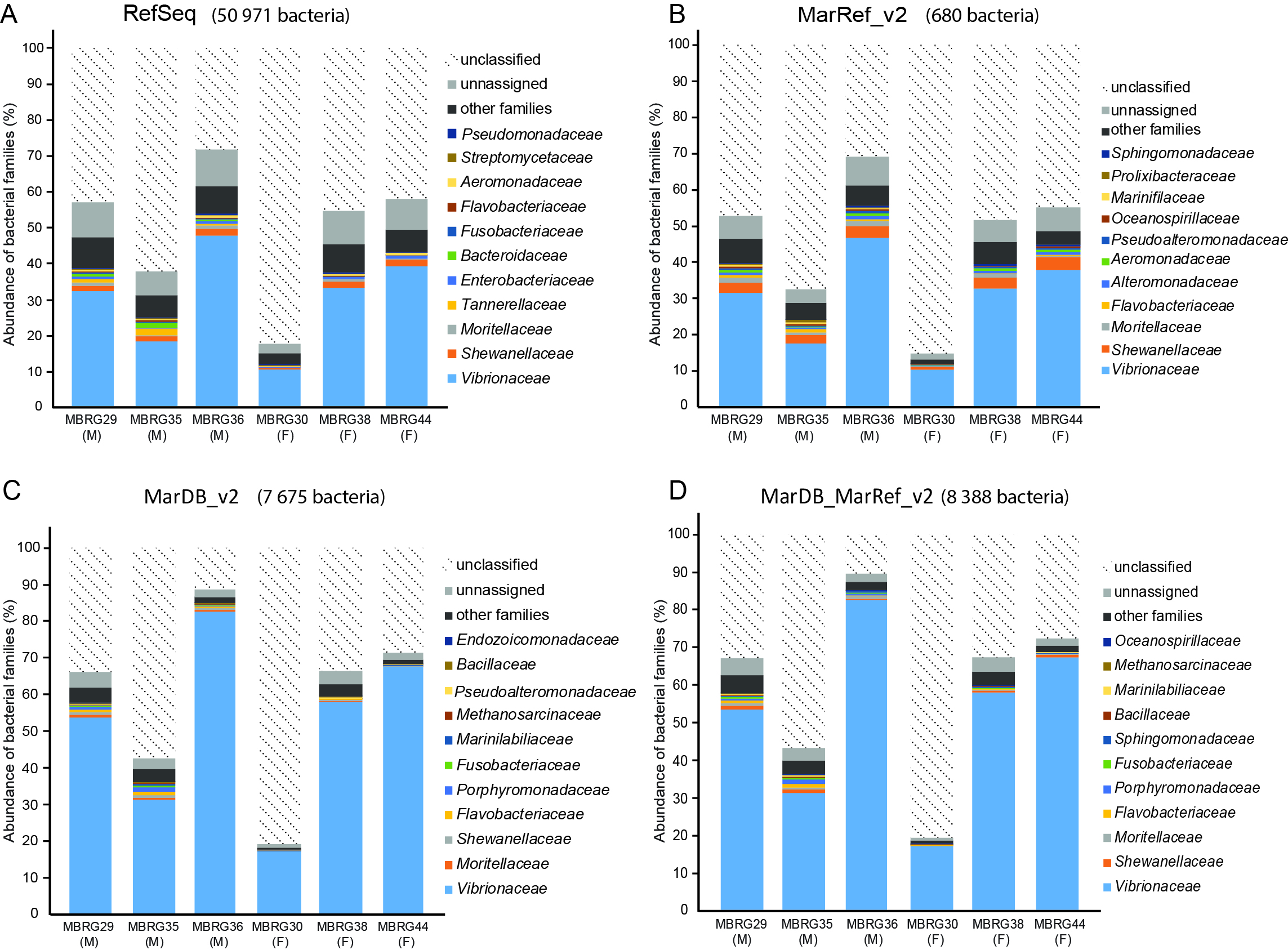

Supplement: Supplementary file 3 — Taxonomic comparison of fecal samples using different databases (RefSeq and the Mar databases) with Kaiju. Graphical representation of the number of detected bacterial families with A) RefSeq, B) MarRef, C) MarDB, and D) combined MarDB with MarRef. (JPG 1932 kb) [file 40168_2019_681_MOESM3_ESM.jpg]

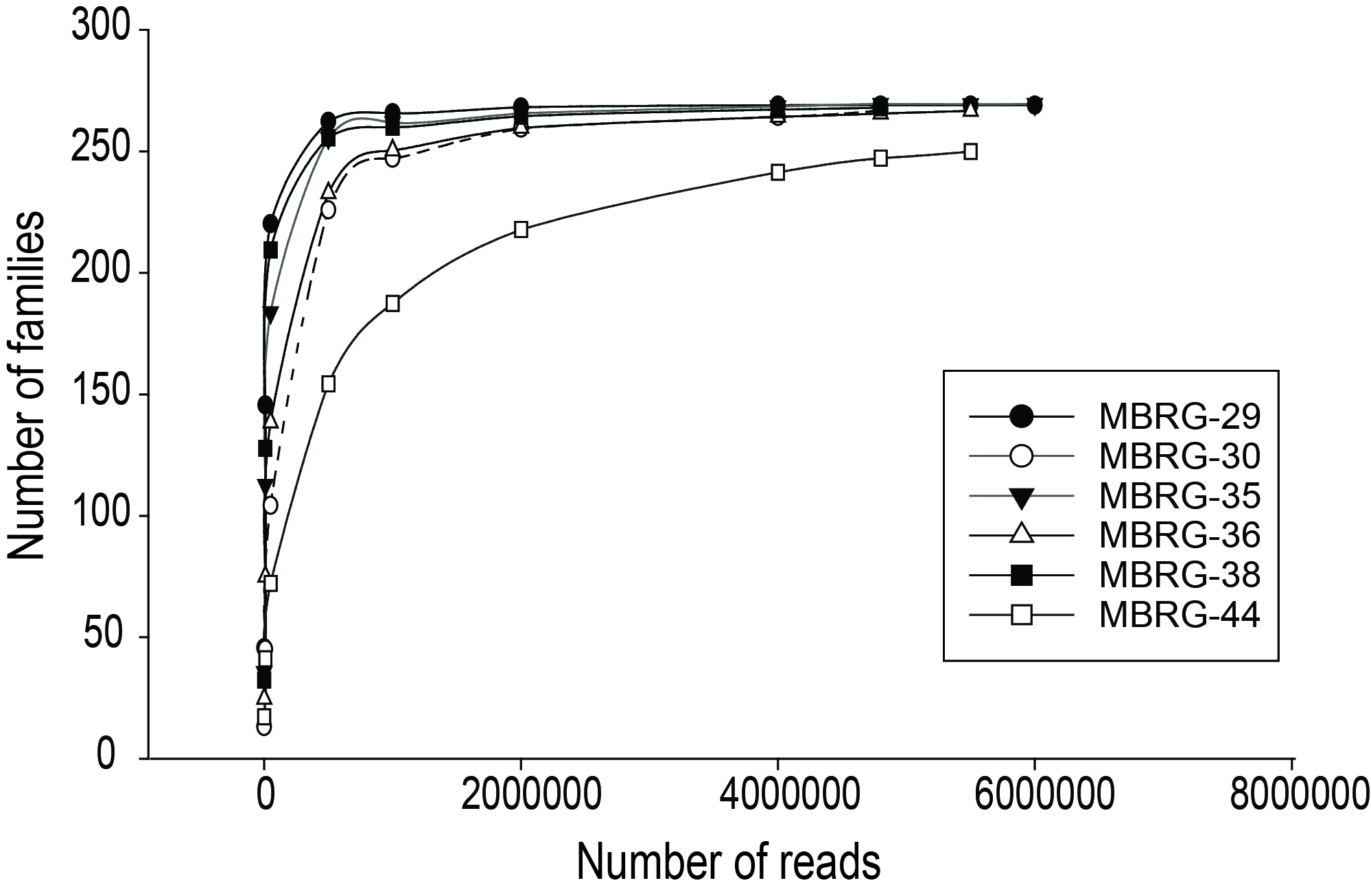

Supplement: Supplementary file 4 — Rarefaction curve analysis of the individual fecal samples. The analysis shows the number of detected families per sample as a function of the number of reads. The six samples include three males (MBRG-29, MBRG-35, MBRG-36) and three females (MBRG-30, MBRG-38, MBRG-44). (JPG 1346 kb) [file 40168_2019_681_MOESM4_ESM.jpg]

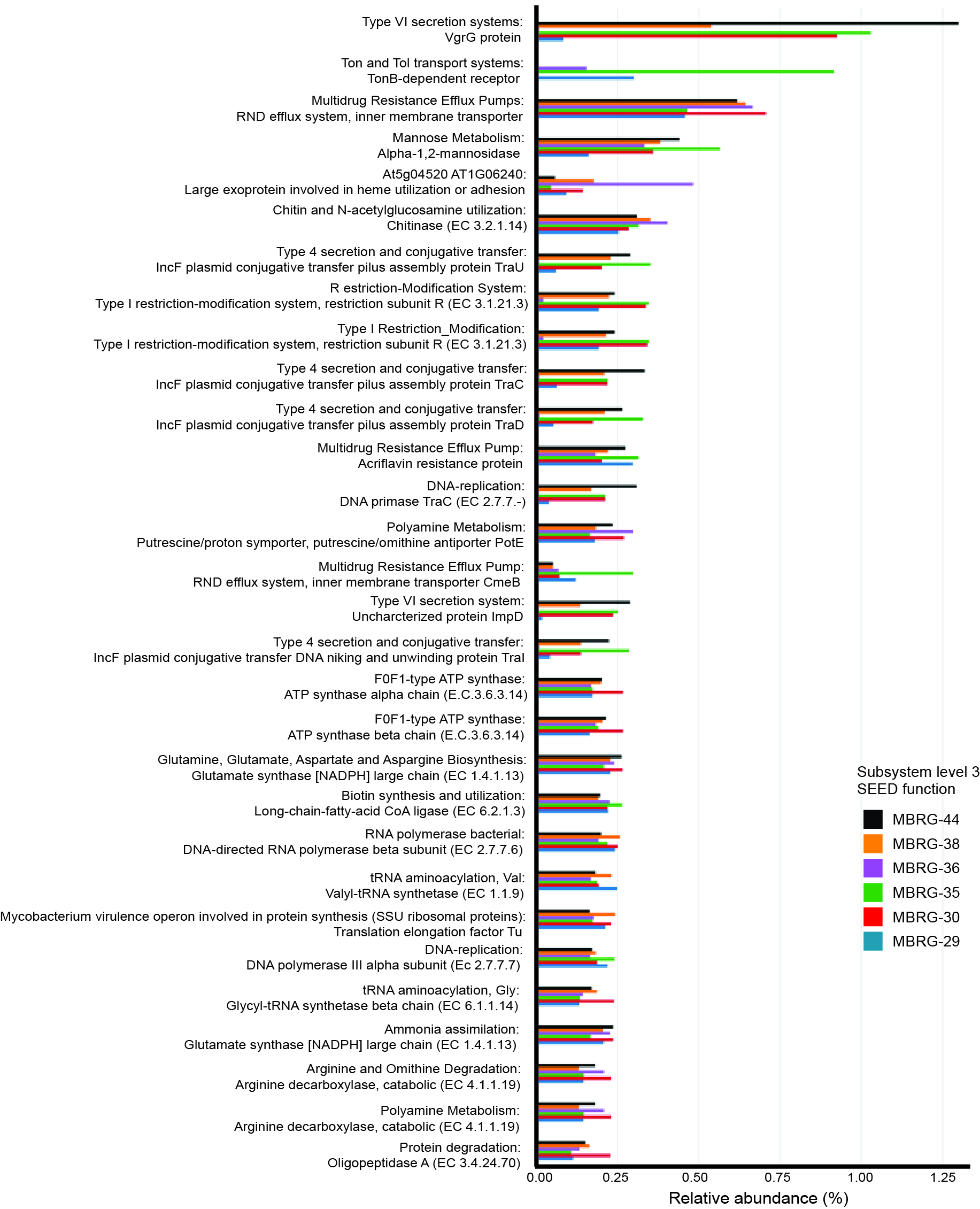

Supplement: Supplementary file 5 — Functional profiling showing the top 30 most occurring SEED functions (including their corresponding subsystem level 3) of all unannotated shotgun sequencing reads from six fecal samples of migrating Atlantic cod. This figure was constructed using SUPER-FOCUS plot. (JPG 1496 kb) [file 40168_2019_681_MOESM5_ESM.jpg]

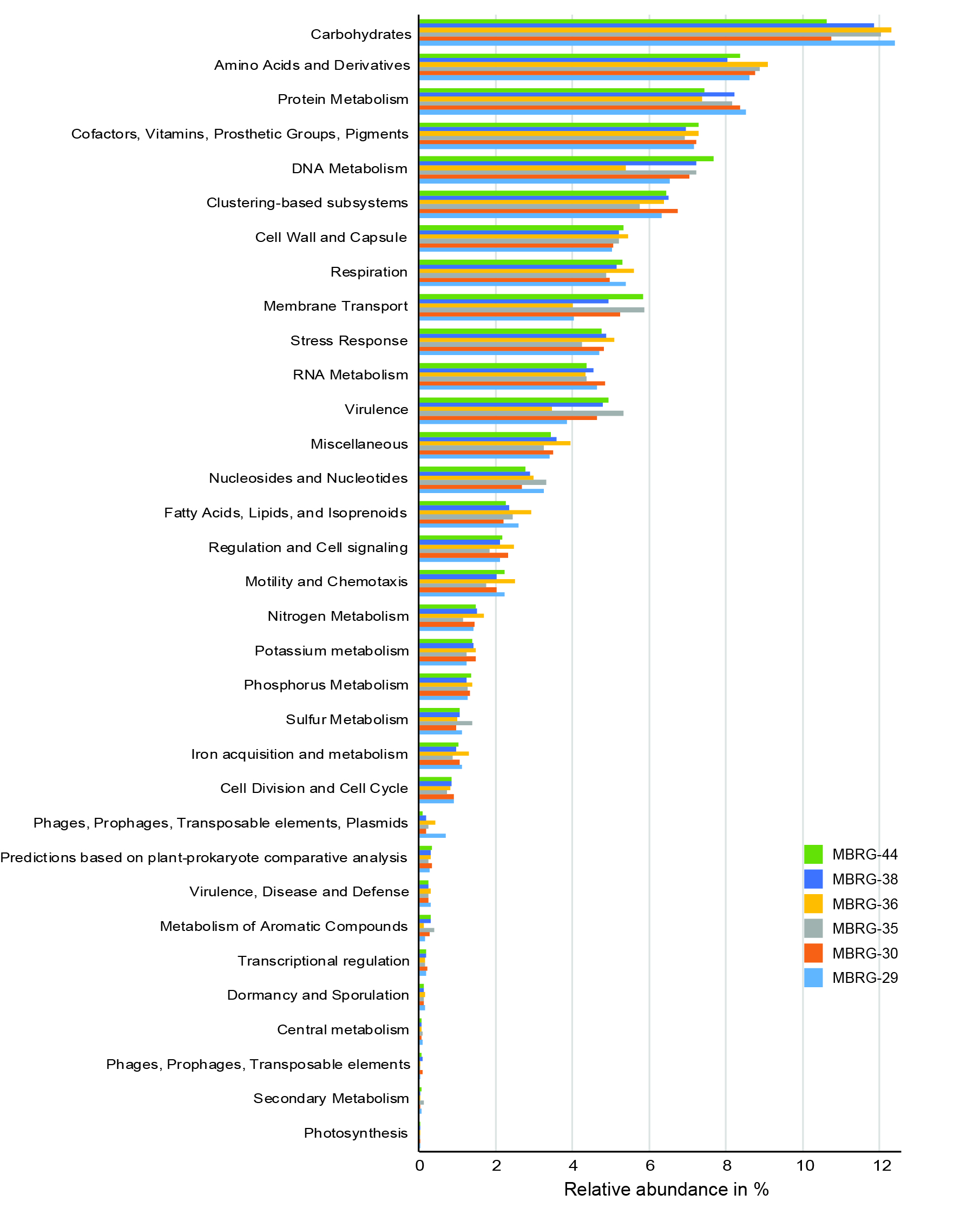

Supplement: Supplementary file 6 — Functional profiling showing the relative abundance of SEED subsystems at level 1 of all unannotated sequencing reads from six fecal samples of migrating Atlantic cod, as derived from SUPER-FOCUS. (JPG 1217 kb) [file 40168_2019_681_MOESM6_ESM.jpg]

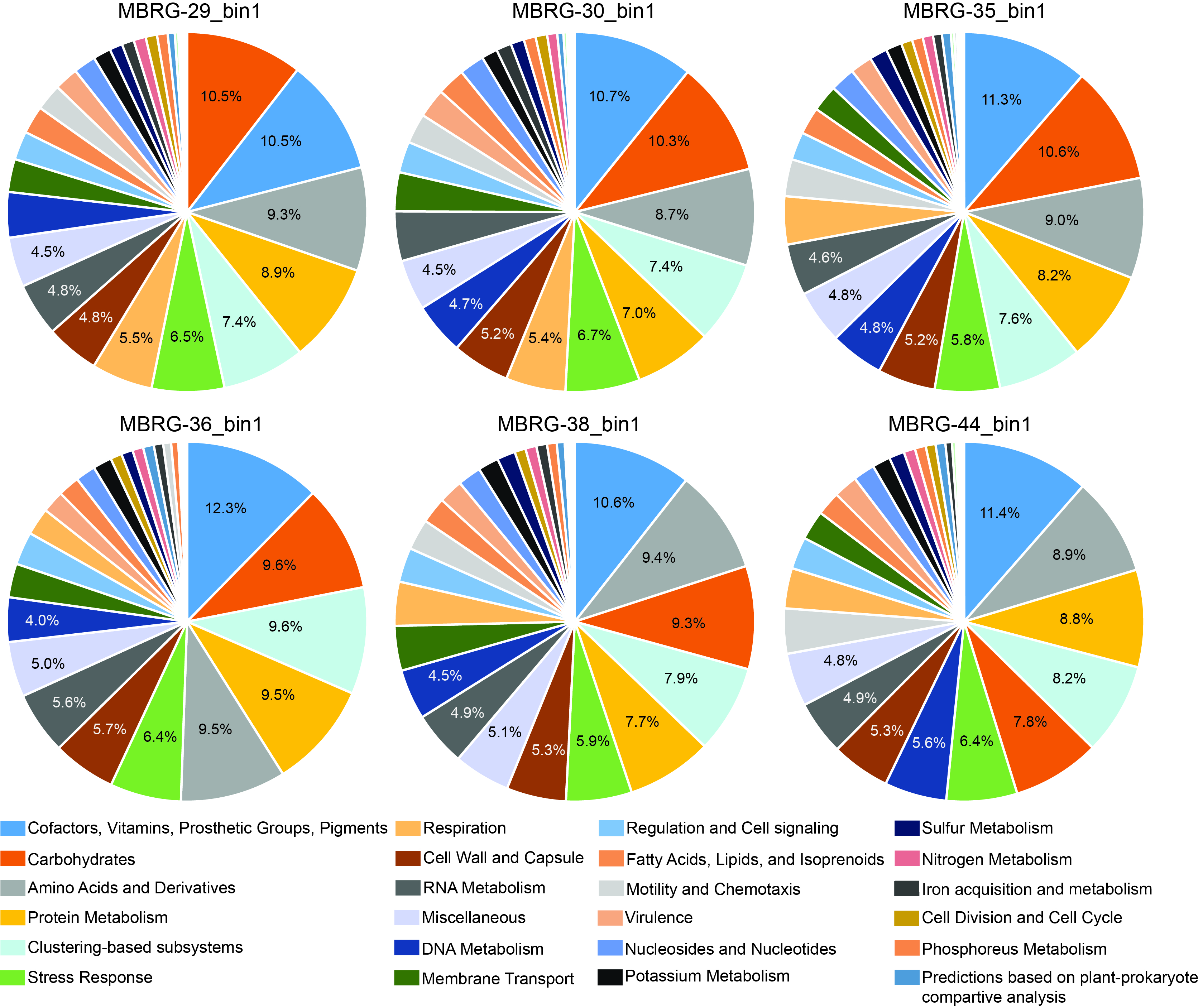

Supplement: Supplementary file 7 — Functional profiling showing the relative abundance of SEED subsystems at level 1 of the most abundant bacteria in the gut of Atlantic cod (i.e., individual profiles of one bin from each sample with the highest relative abundance), as derived from SUPER-FOCUS. (JPG 4081 kb) [file 40168_2019_681_MOESM7_ESM.jpg]

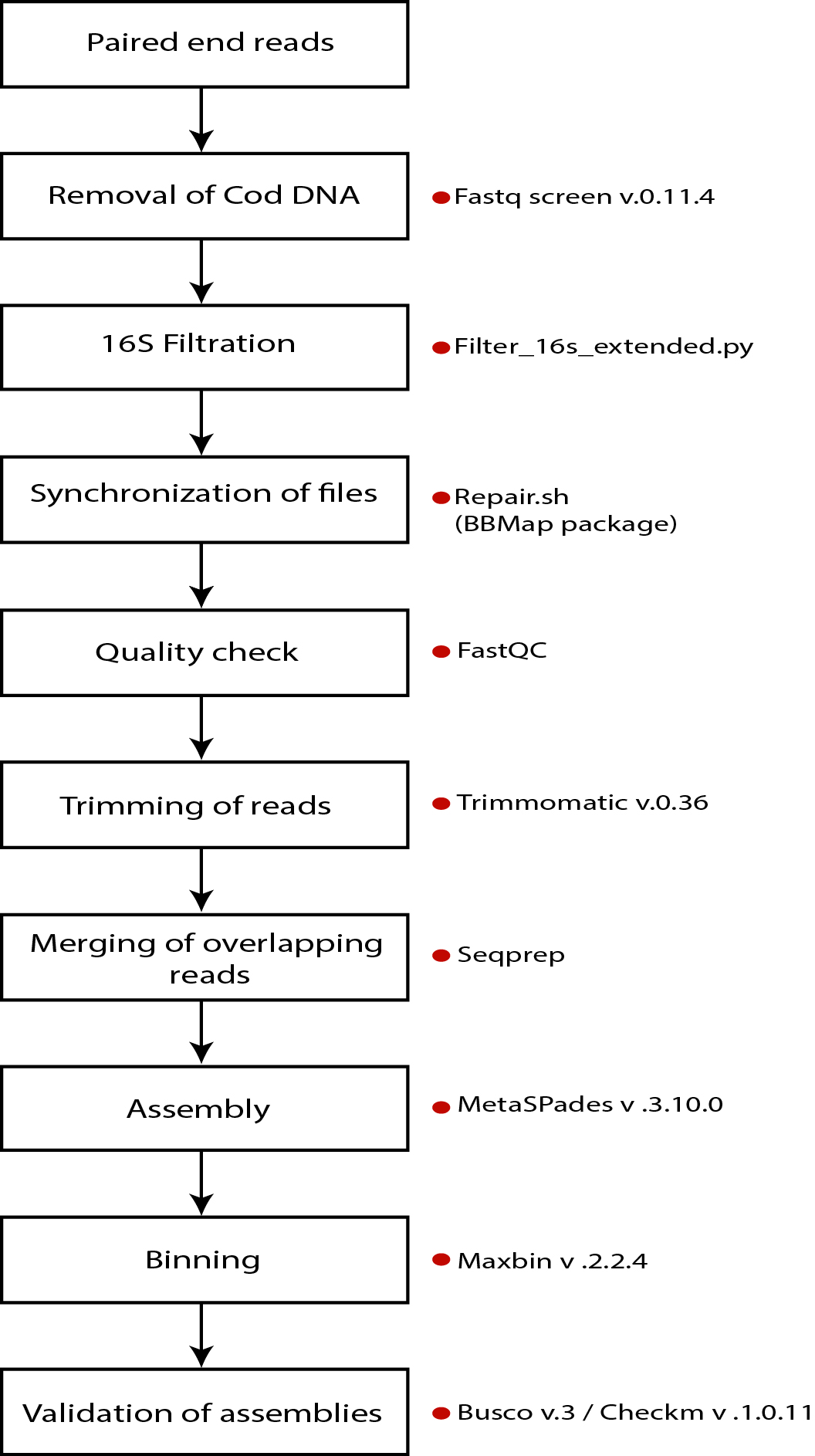

Supplement: Supplementary file 9 — Workflow of the nine steps for recovering metagenome-assembled genomes (MAGs) from six fecal samples of Atlantic cod. (JPG 759 kb) [file 40168_2019_681_MOESM9_ESM.jpg]
